# Patterns and frequency of renal abnormalities in Fanconi anaemia: implications for long-term management

**DOI:** 10.1007/s00467-018-3952-0

**Published:** 2018-04-12

**Authors:** Vijaya Sathyanarayana, Beth Lee, Neville B. Wright, Rui Santos, Denise Bonney, Robert Wynn, Leena Patel, Kate Chandler, Ed Cheesman, Detlev Schindler, Nicholas J. A. Webb, Stefan Meyer

**Affiliations:** 1 Department of Paediatric Nephrology, Royal Manchester Children’s Hospital, Manchester University NHS Foundation Trust, Manchester, UK; 2 Manchester Academic Health Science Centre, Manchester, UK; 3 Department of Paediatric Hematology and Oncology, Royal Manchester Children’s Hospital, Manchester University NHS Foundation Trust, Manchester, UK; 4 Department of Paediatric Radiology, Royal Manchester Children’s Hospital, Manchester University NHS Foundation Trust, Manchester, UK; 5 Department of Paediatric Endocrinology, Royal Manchester Children’s Hospital, Manchester University NHS Foundation Trust, Manchester, UK; 6 Department of Genetic Medicine, St Mary’s Hospital, Manchester University NHS Foundation Trust, Manchester, UK; 7 Department of Paediatric Histopathology, Royal Manchester Children’s Hospital, Manchester, UK; 8 Department of Human Genetics, University of Würzburg, Würzburg, Germany; 9 Young Oncology Unit, The Christie NHS Foundation Trust, Wilmslow Road, Manchester, M20 6XB UK; 10 Paediatric and Adolescent Oncology, Division of Molecular and Clinical Cancer Sciences, Faculty of Biology, Medicine & Health, University of Manchester, Manchester, UK; 11 Academic Unit of Paediatric and Adolescent Oncology, Department of Paediatric Hematology and Oncology, Royal Manchester Children’s and Christie Hospital, University of Manchester, Manchester, UK

**Keywords:** Fanconi anaemia, Renal abnormalities, Long-term follow-up

## Abstract

**Background:**

Fanconi anaemia (FA) is an inherited disease with bone marrow failure, variable congenital and developmental abnormalities, and cancer predisposition. With improved survival, non-haematological manifestations of FA become increasingly important for long-term management. While renal abnormalities are recognized, detailed data on patterns and frequency and implications for long-term management are sparse.

**Methods:**

We reviewed clinical course and imaging findings of FA patients with respect to renal complications in our centre over a 25-year period to formulate some practical suggestions for guidelines for management of renal problems associated with FA.

**Results:**

Thirty patients including four sibling sets were reviewed. On imaging, 14 had evidence of anatomical abnormalities of the kidneys. Two cases with severe phenotype, including renal abnormalities, had chronic kidney disease (CKD) at diagnosis. Haematopoietic stem cell transplantation was complicated by significant acute kidney injury (AKI) in three cases. In three patients, there was CKD at long-term follow-up. All patients had normal blood pressure.

**Conclusions:**

Evaluation of renal anatomy with ultrasound imaging is important at diagnostic workup of FA. While CKD is uncommon at diagnosis, our data suggests that the incidence of CKD increases with age, in particular after haematopoietic stem cell transplantation. Monitoring of renal function is essential for management of FA. Based on these long-term clinical observations, we formulate some practical guidelines for assessment and management of renal abnormalities in FA.

**Electronic supplementary material:**

The online version of this article (10.1007/s00467-018-3952-0) contains supplementary material, which is available to authorized users.

## Introduction

Fanconi anaemia (FA) is an inherited disease associated with variable congenital and developmental abnormalities, bone marrow failure, and cancer predisposition. FA results from defects in the FA/BRCA pathway for DNA interstrand crosslink (ICL) repair, in which multiple proteins encoded by the FA genes interact [1, 2]. Causative mutations have so far been reported in 22 FA genes (*FANCA*, -*B*, -*C*, -*D1*, -*D2*, -*E*, -*F*, -*G*, -*I*, -*J*, -*L*, -*M*, -*N*, -*O*, -*P*, -*Q*, *-R*, *-S*, *-T*, *-U*, -*V*, and *-W)* [1, 3, 4]. At a cellular level, FA is characterized by hypersensitivity to DNA crosslinking agents in terms of cell survival, arrest in the G2 phase of the cell cycle, and chromosomal breakage [5]. The phenotype of FA can be extremely variable [6]. Clinical manifestations commonly include radial ray abnormalities, short stature, microcephaly, and skin pigmentation. Bone marrow failure is very common, and historically, this has been the most relevant clinical manifestation [6]. Over the last 20 years with improved outcome of haematopoietic stem cell transplantation (HSCT) and supportive treatment, the clinical course of FA has changed dramatically, and many individuals with FA now reach their third and fourth decade after correction of haematopoietic failure. For these patients, other problems associated with the underlying genetic defect become increasingly relevant for long-term management. Congenital abnormalities of the kidneys and the urinary tract (CAKUT) are well recognized in patients with FA, with a reported incidence of around 30% [5–8]. However, detailed data with respect to patterns and frequency of abnormalities involving the kidneys are sparse. In addition, implications of renal abnormalities for the long-term management have not been fully assessed, and detailed guidelines for the diagnosis and management of renal abnormalities in FA have not been formulated. These should consider the inherited DNA repair defect and therefore minimize the use of X-rays, because of the potential harm caused in chromosomal instability syndromes such as FA [9]. To address the relevance of patterns and frequency of renal abnormalities for long-term follow-up, we reviewed the incidence and patterns, and the clinical course of patients with FA in our centre together with available genetic data to aid the formulation of guidelines for the management of FA-associated renal problems.

## Materials and methods

All patients diagnosed with FA based on clinical findings and demonstration of characteristic increased cellular mitomycin C sensitivity, in most cases complemented by mutational analysis, treated in our centre over the last 25 years were included. We retrospectively analyzed imaging and biochemical laboratory investigations at diagnosis and during their clinical course, including pre-, inter-, and post-HSCT and at long-term follow-up. Patients were grouped for presence and severity of FA-associated clinical features, including haematological, skeletal, central nervous system (CNS), and other abnormalities. Patients were classed as having a mild phenotype when in addition to haematological abnormalities at diagnosis only subtle microcephaly and short stature were present and no obvious radial ray abnormalities. Classical phenotype included radial ray abnormalities and bone marrow failure with typical skin pigmentations, short stature, and microcephaly. Patients were considered to have a severe phenotype when, in addition to the above, they exhibited extreme short stature and abnormalities also seen associated with the VACTER-L spectrum (vertebra, cardiac and trachea-esophageal malformations, limb malformations), and/or CNS, cardiac or anorectal abnormalities were present.

## Results

### Patients

Thirty patients with FA (16 females) were included. The median age at diagnosis was 5 years (range 5 months to 10 years). Phenotypic patient characteristics were considered mild in four cases, classic in 21, and severe in five cases (Table 1). A detailed description of patients is provided in Supplemental Table 1.

**Table 1:** Clinical and genetic characterization of Fanconi anaemia patients studied

	No	Gene mutation	Renal abnormalities
Severe phenotype			
Multiple congenital abnormalities Bone marrow failure evident < 5 years of age Extreme short stature	5	*FANCD1/BRCA2 - 1* *FANCA - 1* *FANCF - 2* Undetermined - 1	4
Classic phenotype			
Bone marrow failure Microcephaly radial ray abnormalities Short stature and clinically subtle other abnormalities	21	*FANCA - 13* *FANCG - 3* *FANCI - 1* Undetermined *-* 4	9
Mild phenotype			
Subtle physical abnormalities Bone marrow failure or hypoplasia	4	*FANCD2 - 1* *FANCA - 2* Undetermined - 1	1

### Imaging

All patients were investigated with renal ultrasound scan at presentation or early in the clinical course. Abnormalities were detected in 14 patients and included six dysplastic, three pelvic, two malrotated kidneys, and one each of crossed fused ectopia, horseshoe, and multi-cystic kidney (Supplemental Table 1, Supplemental Figure 1). A micturating cystourethrogram (MCUG) was performed in two male patients, one with a left pelvic kidney and hydronephrosis in the right kidney, and the other with bilateral renal dysplasia, which demonstrated vesicoureteral reflux (VUR) grade V and II (patient 3 and 23, Supplemental Figure 2A). DMSA (dimercaptosuccinic acid) scan was performed in three patients for assessment of split function, which demonstrated reduced differential renal function in two (patient 22 and 23, Supplemental Figure 2B). All of these abnormalities were managed conservatively. Four sibling sets were included in our study cohort, of which one pair had small dysplastic kidney in one sibling, which was further assessed by magnetic resonance (MR) imaging (patient 10, Supplemental Figure 3A and B). While most patients with severe phenotype had renal abnormalities, these were also detected patients with classic FA or with an otherwise subtle phenotype. There was no obvious correlation between kidney abnormality and mutational genotype.

### Infections

Six patients had proven urinary tract infections (UTIs) in early childhood, requiring antibiotic treatment followed by prophylaxis. All patients with UTIs had abnormal anatomy on imaging, with MCUG findings documenting VUR in two male patients (patient 3 and 23), and reduced asymmetric kidney function on DMSA scanning in one patient (patient 22). The other three patients with UTIs had small dysplastic kidneys and a recto-vaginal fistula, horseshoe kidney, and fused ectopic kidneys (patients 1, 2, and 4).

### Renal function at diagnosis

At diagnosis, chronic kidney disease (CKD) was noted in two cases. One case with severe phenotype and death from rapid leukaemic transformation had renal dysplasia on ultrasound scan with an estimated glomerular filtration rate (eGFR) of less than 15 ml/min/1.73m^2^ at presentation (patient 3) [10]. The second case, also with severe phenotype including complex heart disease, had a left pelvic kidney with an eGFR of 30 ml/min/1.73m^2^. The level of renal function improved with management of the infant’s complex clinical needs including gut and heart surgery (patient 2).

### Renal complications during HSCT

Of the 24 patients undergoing HSCT using sibling or matched unrelated cord blood or bone marrow, acute kidney injury (AKI) post-HSCT was seen in three patients (14%). All three patients had KDIGO (kidney disease improving global outcome) stage 1 AKI with rise > 1.5 × baseline serum creatinine. Two patients had dysplastic kidneys and one had normal kidneys on ultrasound. In all three patients, AKI was suspected to be related to toxicity from ciclosporin administration, despite absence of documented high trough levels. Renal function improved in all three after dose reduction or withdrawal of ciclosporin, and none required renal replacement therapy.

### Outcome and follow-up

One case with severe phenotype and CKD diagnosis from extreme renal dysplasia and severe VUR died of leukaemic transformation at the age of 2 years (patient 3) [10]. Two children died during HSCT of acute transplant-related complications with multi-organ failure, one of whom had a crossed fused ectopic kidney (patients 4 and 7). Seven children, including two who have undergone HSCT, are currently followed up for less than 5 years. Impaired renal function with CKD stage III was present in one child with severe phenotype before HSCT. This has to date been managed conservatively. All six others have normal renal function, including one child, who had one severely dysplastic non-functional kidney removed. On histological examination, this showed complete disorganization of the renal parenchyma with extensive fibrosis and partly cystically dilated tubules cuffed by primitive mesenchyme (patient 22, Supplemental Figure 4). Follow-up data longer than 5 years (range 5 to > 25 years) were available for 23 cases, of which three patients have not undergone HSCT, including a middle-aged female diagnosed in childhood with classic phenotype FA with a pelvic kidney and only subtle haematological abnormalities (patient 18) [11], and two are awaiting HSCT. During long-term follow-up after diagnosis and HSCT, three patients were noted to have biochemical evidence of CKD (eGFR 20–40 ml/min/1.73m^2^). All three had small dysplastic kidneys (patients 10, 14, and 21). All 30 patients had normal blood pressure at presentation and at follow-up.

## Discussion

Renal and urinary tract abnormalities in FA are common, with previous studies suggesting that around one third of patients have structural renal abnormalities [12, 13]. Our analysis identifies a prevalence of 50%, confirming the high frequency of earlier observations in what is, to our knowledge, the largest cohort specifically reporting renal aspects of FA. The patterns of abnormalities detected point to perturbed normal ascent of the embryonic kidney in FA and imply a potential role for the FA pathway in particular during early embryonal stages of kidney development. Further support for this hypothesis comes from a recent study reporting brca2/fancd1 deficiency in zebrafish leading to perturbed pronephros development [14]. As in previous studies [12], we did not identify an obvious correlation between genotype and presence of kidney abnormalities. Given the high incidence of renal abnormalities, anatomical and functional evaluation is important at diagnostic workup of FA. While obvious and significant renal dysfunction is uncommon at diagnosis, and in our cohort in all cases was associated with a severe phenotype, the high incidence of UTIs in our cohort, and findings of abnormal functional imaging suggests that children with FA may have compromised but compensated renal function. Published case reports support our findings that a minority of FA patients have severe renal problems at diagnosis, and CKD can be a serious complication of FA [15]. For baseline renal assessment in FA, as summarized in Table 2, we would recommend ultrasound imaging accompanied by regular biochemical monitoring. Decisions with respect to additional radiological investigations would need to be taken on an individual basis. Mindful of the potential higher sensitivity to radiation, imaging could alternatively be carried out using MR (Supplemental Figure 3). Although there is no published experience specifically in FA, MR-urography has been used by us and others for reflux and assessment of renal function, avoiding ionizing radiation exposure [16, 17]. In our cohort, renal impairment at diagnosis was seen in children with severe phenotype including early severe haematological complications. Early renal impairment might have some prognostic significance in general for FA that would need to be confirmed in larger patient groups. Only three patients in our study encountered renal complications acutely during HSCT, which is less than other FA patient cohorts described in the literature [18]. While this might just reflect our selected patient population, we conclude that overall acute renal complications during HSCT are manageable in FA, but can also affect patients with normal kidneys on imaging. Renal sequelae have not specifically been studied with respect to the long-term outcome in FA, including non-transplanted patients and post-HSCT [19]. Data from our cohort suggest that renal dysfunction post-HSCT might be more common in FA patients than reported for children transplanted for other indications [20] and suggest that the incidence of renal dysfunction post-HSCT might increase with age in some cases of FA. However, most children maintained normal kidney function, and longer follow-up evaluation of larger groups of FA, and also of non-FA children with respect to renal health after HSCT, will be necessary to assess comprehensively the incidence and causation of renal dysfunction. Importantly, in our cohort, this also affected patients with normal renal anatomy on ultrasound imaging and normal kidney function at diagnosis of FA. An intrinsic increased sensitivity to genotoxic stress in kidneys of FA patients might underlie this observation, in particular as this has been observed in mice with FANCD2/FANCI-associated nuclease (FAN1) disruption, and renal sensitivity to genotoxic stress has been reported in response to cross-linking agents and other substances, such aristolochic acid [21, 22]. More detailed prospective analysis of kidney function, also prospectively including urinary protein excretion pre- and post-HSCT, will be able to clarify biology and clinical relevance for children with FA.

**Table 2:** Pragmatic approach to assessment and management of renal manifestations of Fanconi anaemia

Diagnosis	Pre-HSCT	Long-term follow-up
Assess renal function and biochemistry	Assess renal function and biochemistry	Assess renal function and biochemistry 6 monthly
Ultrasound scan of urinary tract If abnormal consider individual need for further imaging, e.g. MR urography, DMSA scan Enhanced surveillance for UTI if CAKUT detected	Enhanced surveillance for UTI if CAKUT detected Consider use of antibiotic prophylaxis, in particular with VACTER-L-type abnormalities, complex abnormalities, and evidence of reflux	
Blood pressure monitoring	Blood pressure monitoring	Blood pressure monitoring

*CAKUT* congenital abnormalities of kidneys and the urinary tract, *DMSA* dimercaptosuccinic acid, *HSCT* haematopoetic stem cell transplant, *MR* magnetic resonance, *UTI* urinary tract infection

Renal disease is clearly important for the management of FA at diagnosis and early clinical course and during HSCT. Furthermore, renal function is also relevant for management of later complications in FA, such as other malignancies. With these points in mind, we suggest a few pragmatic guidelines for renal assessment of FA, as outlined in Table 2. Every patient should have imaging by ultrasound scan; further imaging may be necessary on an individual basis, but X-ray exposure should always be considered, and additional investigations justified. Close monitoring for UTIs in young children with a low threshold for antibiotic intervention and lifelong monitoring for renal function is important for long-term follow-up in FA.

## Electronic supplementary material

Supplemental Figures (PDF 1029 kb)

Supplemental Table 1 (DOCX 35 kb)
