# Supplementary material for: Patterns and frequency of renal abnormalities in Fanconi anaemia: implications for long-term management
Source: Pediatr Nephrol. 2018 Apr 12;33(9):1547–51. doi: 10.1007/s00467-018-3952-0 (PMC6061664; doi:10.1007/s00467-018-3952-0)
Supplement: Supplementary file 1 — (PDF 1029 kb) [file 467_2018_3952_MOESM1_ESM.pdf]

Supplemental Figure 1

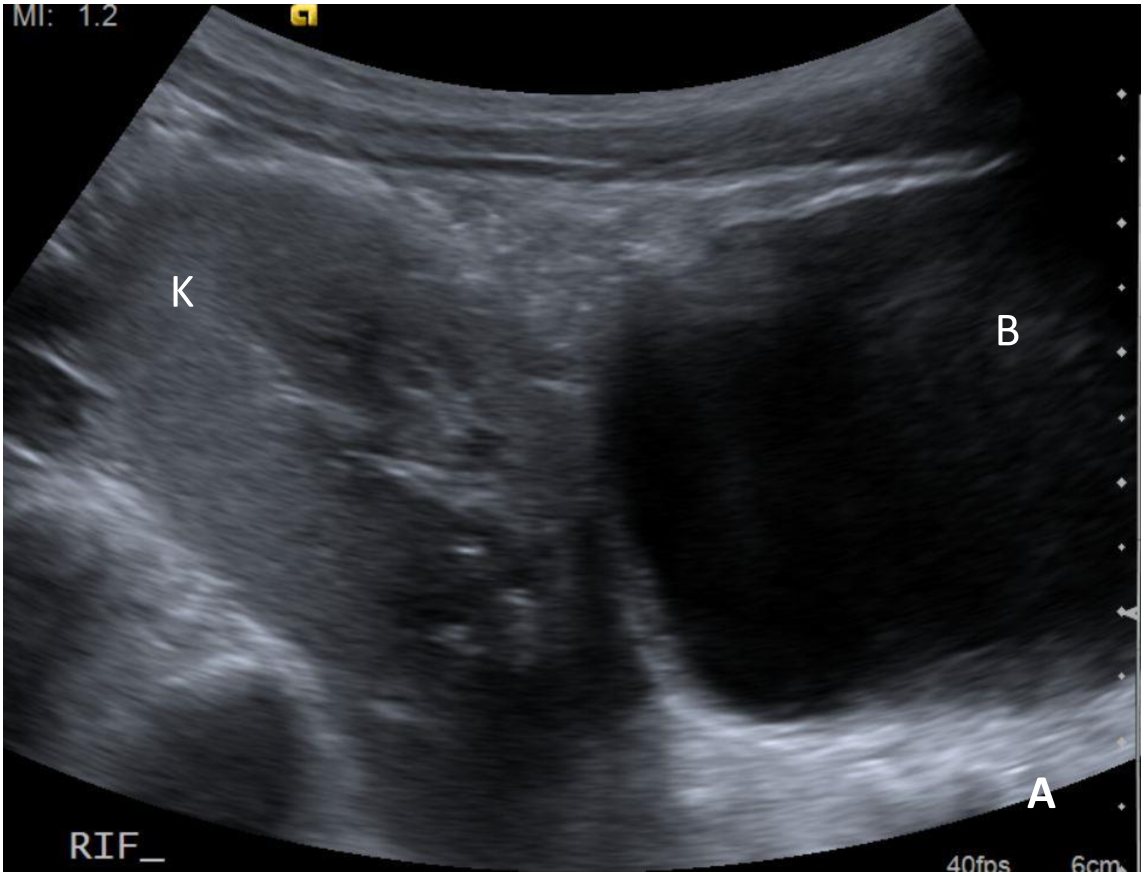

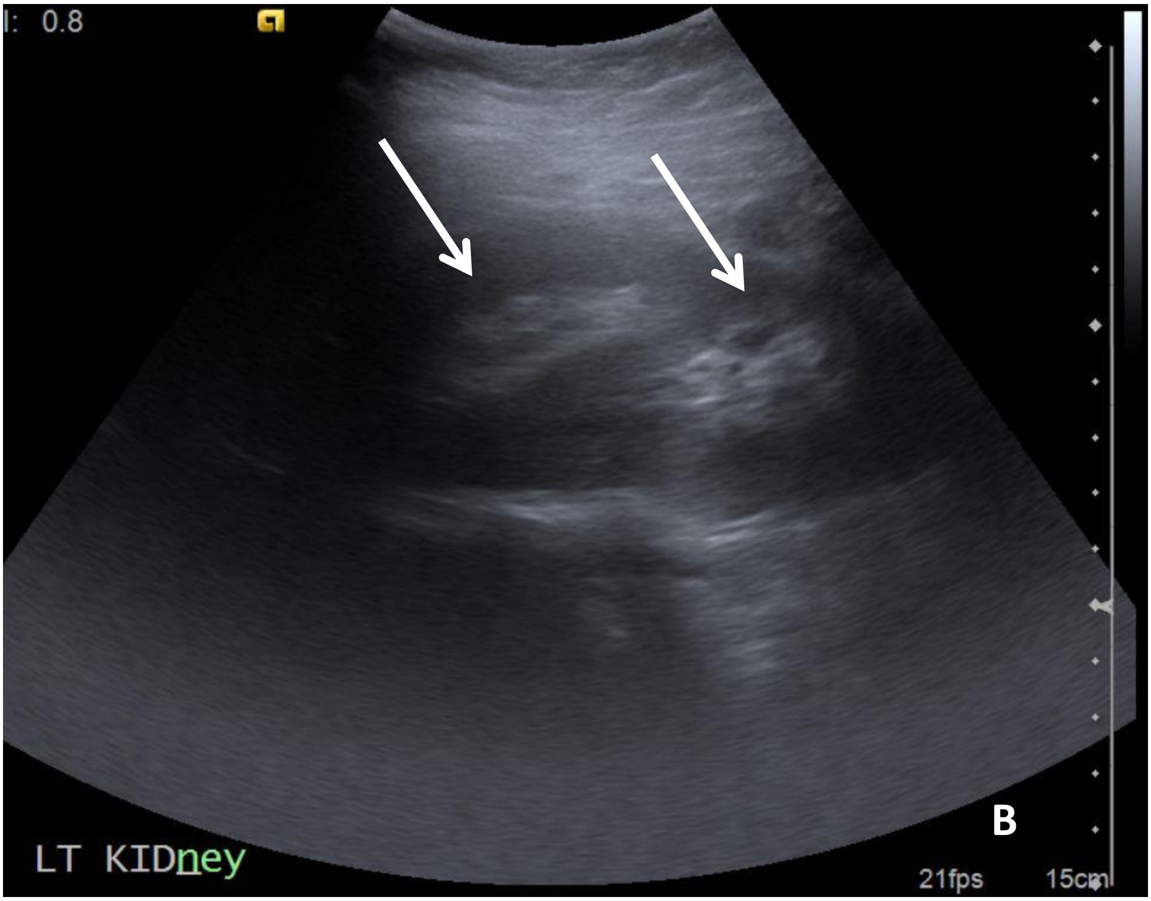

Supplemental Figure 2

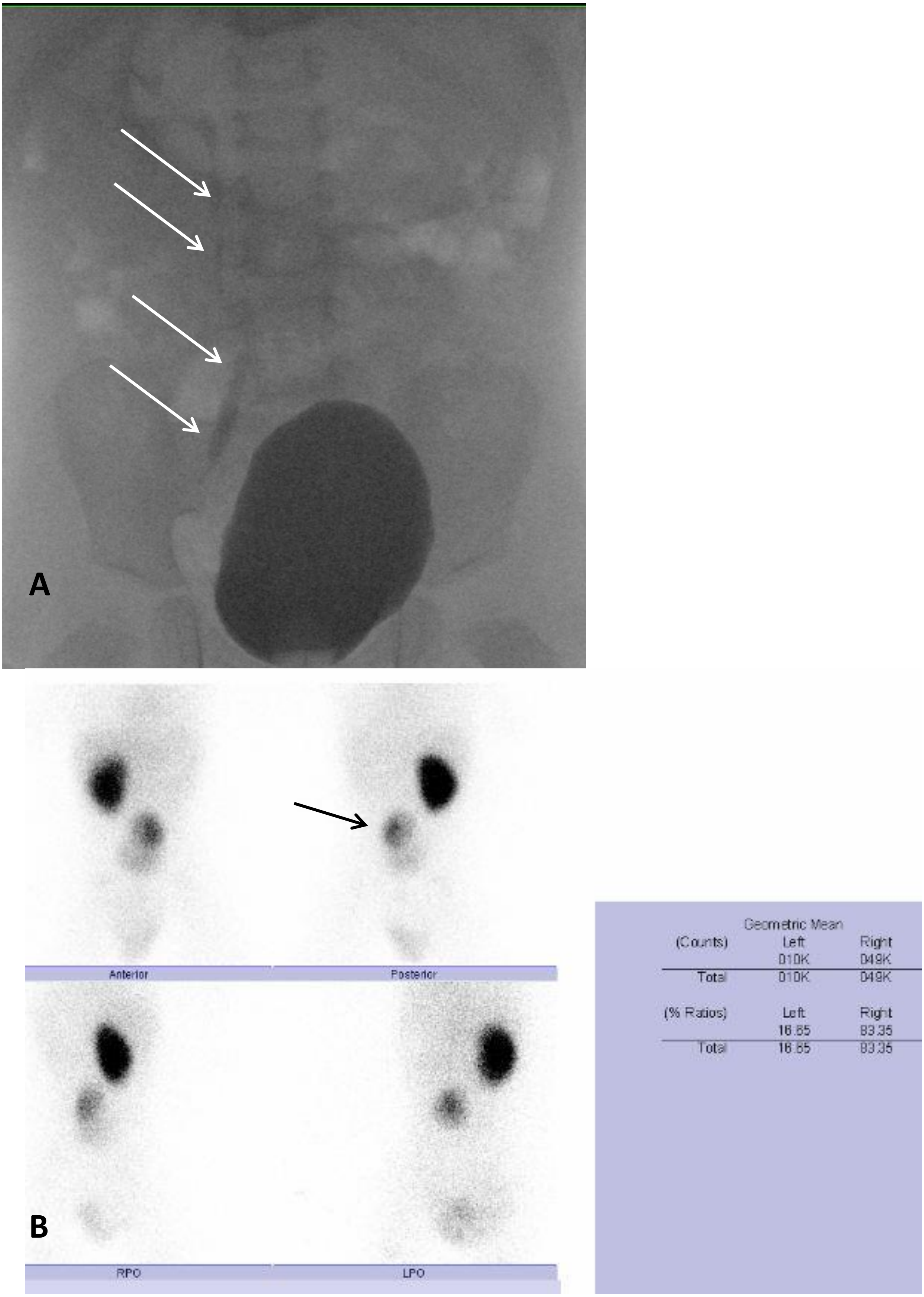

Supplemental Figure 3

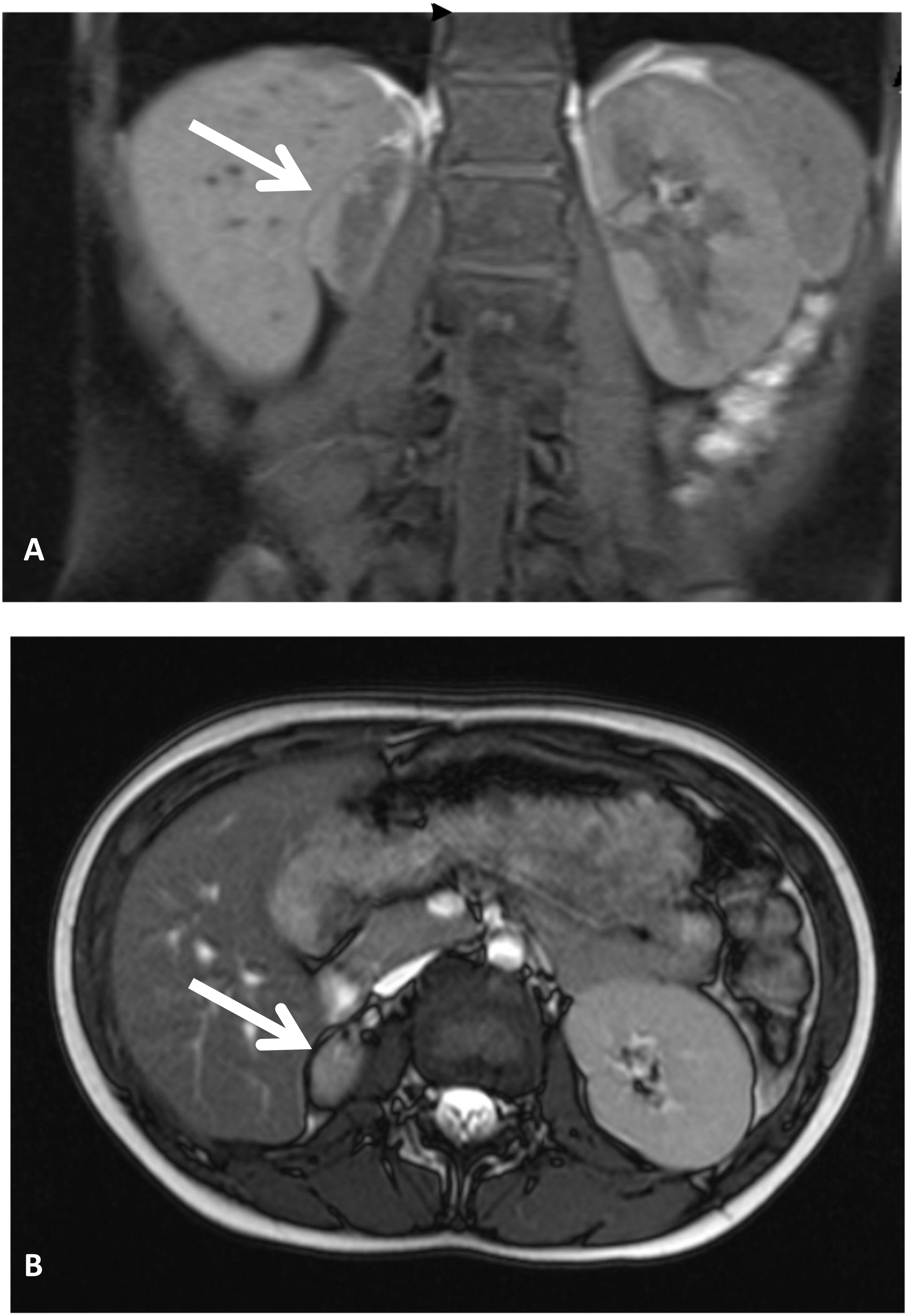

Supplemental Figure 4

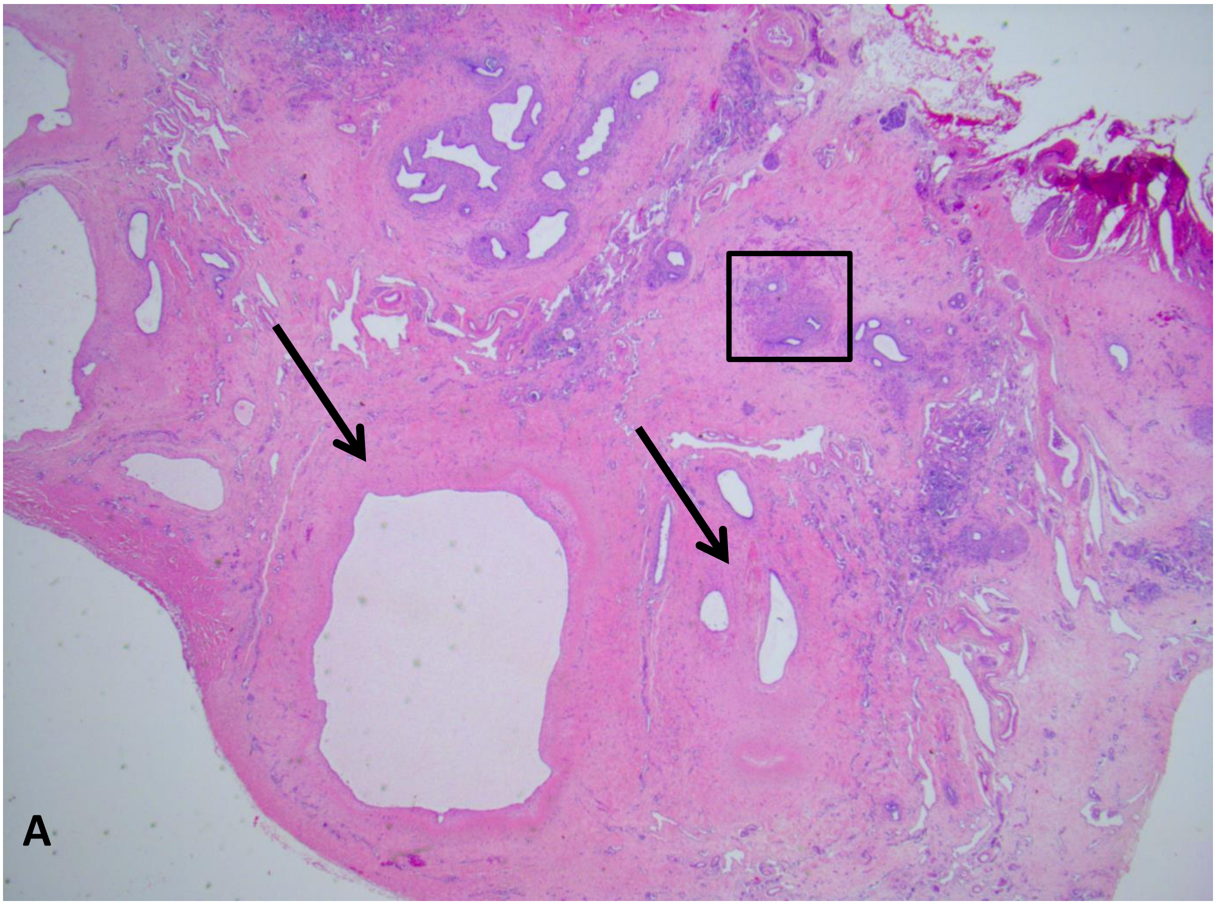

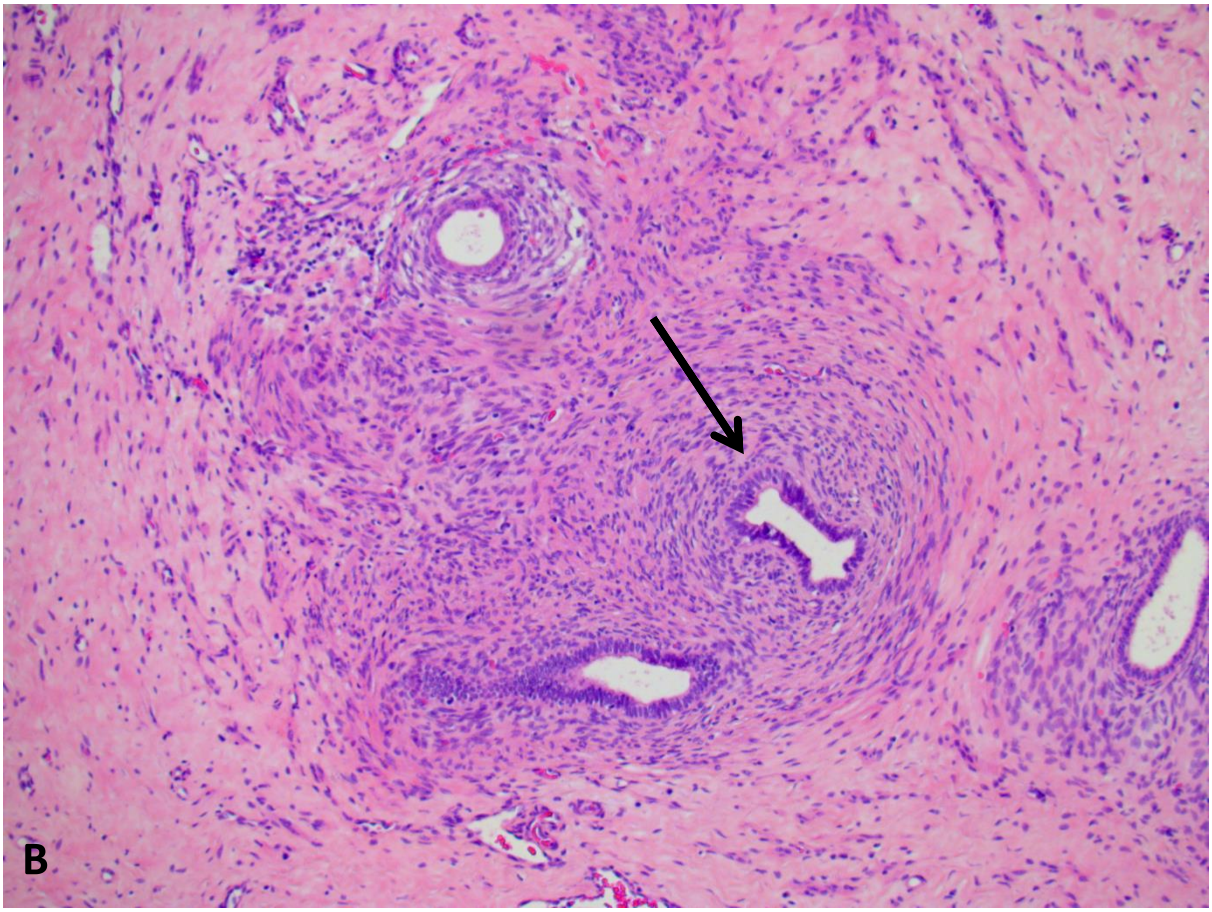

Figure legends to supplemental figures:

Supplemental Figure 1: Ultrasound findings in individuals with Fanconi anaemia **.** **A:** Pelvic kidney (K) in close proximity to the filled Bladder (B). **B:** Fused ectopic Kidney with two collecting systems (arrows).

Supplemental Figure 2: A: Micturating cysto-urethrogram (MCUG) demonstrating vesico-ureteral reflux (arrows) in a patient with FA. **B.** DMSA scan showing decreased uptake and therefore impaired function of an ectopic dysplastic kidney.

Supplemental Figure 3: A: Coronal T1 FS sequence shows an atrophic right side kidney marked (arrow). Normal size left kidney B: Axial T1 sequence. Small kidney diameter (arrow) of left dysplastic kidney.

Supplemental Figure 4: Histology of dysplastic kidney associated with FA **A:** Complete disorganisation of the renal parenchyma with extensive fibrosis (x40) **. B**: Dysplastic tubules are cuffed by primitive mesenchyme (x100), and several are cystically dilated (arrows).
