# Supplementary material for: Patterns and frequency of renal abnormalities in Fanconi anaemia: implications for long-term management
Source: Pediatr Nephrol. 2018 Apr 12;33(9):1547–51. doi: 10.1007/s00467-018-3952-0 (PMC6061664; doi:10.1007/s00467-018-3952-0)
Supplement: Supplementary file 2 — (DOCX 35 kb) [file 467_2018_3952_MOESM2_ESM.docx]

| **Patient** | **Sex** | **Clinical Phenotype at diagnosis** | **Ethnicity** | **C/S** | **Mutated FA gene** | **Age of Diagnosis** | **Clinical course – general** | **Renal assessment and follow up** | | | |
| --- | --- | --- | --- | --- | --- | --- | --- | --- | --- | --- | --- |
|  |  |  |  |  |  |  |  | ***USS Other*** | | ***Function at presentation at length of FU*** | |
| 1 | **F** | - Congenital complex heart condition - Anal atresia with rectovaginal fistula - Short stature - Failure to thrive - Hypoplastic thumb | Asian | Y | *FANCF* | 4 months |  | Bilateral dyspl. kidneys | Recurrent UTIs as infant | GFR 30mls/  min/  1.73m^2^ | 10 months  borderline elevated improved serum creatinine  GFR 30-40 mls/min/  1.73m^2^ |
| 2 | **M** | - Extreme short stature - Bilateral thumb abnormality - BMF age 2 | Asian | Y | *FANCF* | 9 months | HSCT age 3 | Horse shoe kidney | UTI in infancy  DMSA normal | normal | 7 years  normal renal function |
| 3 | **M** | - IUGR - Microcephaly - Hypermobile left thumb - Midfacial hypoplasia - Inner ear deafness - Myelodysplsia and development of AML - Imperforate anus | White British | N | *BRCA2/*  *FANCD1* | 18 months | Died age 2 of refractory AML | Bilateral dysplastic kidneys | UTI in infancy MCUG (Bilateral grade 5 VUR) | GFR <15 mls/  min/1.73 m ^2^ | 6 months, died of acute myeloid leukemia |
| 4 | **F** | - Short stature - Congenital heart disease - FTT - Gastrostomy feeds - Small pituitary gland - Bone marrow failure | Asian | N | *undetermined* | 8 years | Correction of CHD in infancy  BMF age 9  HSCT age 10  Died of transplant complications | Right kidney fused with the lower pole of the left kidney | UTI in infancy | normal | 5 years  normal renal function |
| 5 | **F** | - Complex upper limb & radial - abnormalities - Microcephaly - Pituitary Endocrinopathy with hypogonadotropic gonadism & hypothyroidism - Hearing problems - Extreme short stature - BMF | White  British | N | *FANCA* | 3 years | HSCT age 5  Growth Hormone and estrogen  replacement  Hearing aids | Both kidneys normal |  | normal | 19 years  normal renal function |
| 6 | **F** | - Subtle thumb abnormality - Extensive Café au lait spots - BMF - Short stature | Asian | Y | *FANCA* | 7 years | Blood product supplements,  HSCT for BMF imminent age 9 | Both kidneys normal |  | normal | 3 years  normal renal function |
| 7 | **F** | - Microcephaly - Radial abnormalities - BMF - Extreme short statue | Arabic | Y | undetermined | 6 years | Severe BMF with leukaemic transformation  Prolonged androgen treatment.  Died of transplant related complications. | Both kidneys normal |  | normal | 3 months, died of transplant related complications |
| 8 | **M** | - Microcephaly - Bone marrow hypoplasia - Short stature | White British | N | *FANCA* | 18 months | HSCT age 3 | Left pelvic kidney  Normal right kidney |  | normal | 15 years  normal renal function |
| 9 | **M** | - Microcephaly - Subtle unilateral thumb hypoplasia - Short stature - Hypothyroidism - BMF | Asian | Y | *FANCA* | 5 years | HSCT age 7  Thyroid hormone substitution | Both kidneys normal |  | normal | 11 years  Normal renal function |
| **10 S1** | **M** | - Microcephaly - Short stature - Subtle thumb hypoplasia - Hypoplastic left kidney - BMF | Asian | Y | *FANCA* | 5 years | BMF  HSCT age 10 | Right dyspl. kidney  Normal left kidney | MRI abbdomen | AKI  post HSCT | 6 years, mildly elevated serum creatinine  eGFR 20-40 mls/min/1.73m^2^ |
| **11 S2** | **M** | - Bone marrow hypoplasia age 3 - Short stature | Asian | Y | *FANCA* | 2 years (sibling) | HSCT awaited | Both kidneys normal |  | normal | 6 years  normal renal function |
| 12 | **F** | - microcephaly - BMF - Short stature | White British | N | *FANCA* | 4 years | Severe BMF  HSCT age 5 | Both kidneys normal |  | normal | 15 years  normal renal function |
| 13 | **M** | - duplex thumb - bone marrow failure - short stature | Asian | Y | *FANCG* | 4 years | BMF  HSCT awaited | Bilateral dysplastic kidneys |  | normal | 6 years  normal renal function |
| **14 S1** | **M** | - Mild thumb hypoplasia - Short stature - Microcephaly | Asian | Y | *FANCA* | 5 years | HSCT age 7  Hypothyroidism  Pollicisation of index finger | Both kidneys normal |  | AKI  post  HSCT | 17 years  Borderline elevated serum creatinine  eGFR 20-40 mls/min/1.73m^2^ |
| **15 S2** | **F** | - Short stature - Extensive café au lait spots - mild microcephaly - BMF | Asian | Y | *FANCA* | 1 year (sibling) | HSCT age 8  Hypothyroidism | Both kidneys normal |  | normal | 17 years  normal renal function |
| **16 S1** | **F** | - Microcephaly - Subtle thumb hypoplasia - Short stature - BMF | Asian | Y | *FANCG* | 6 years | HSCT age 8 | Both kidneys normal |  | normal | 5 years  normal renal function |
| **17 S2** | **M** | - Microcephaly - BMF - Short statue | Asian | Y | *FANCG* | 5 years (sibling) | HSCT age 7 | Both kidneys normal |  | normal | 5 years  normal renal function |
| 18 | **F** | - Microcephaly - Bone Marrow hypoplasia - Bilateral thumb aplasia - Diabetes - Ectopic anus & - Short stature | White British | N | *undetermined* | 3 years | Pollicisation age 3  Stable haematopoiesis age 50  Supportive treatment for diabetes | Absent left kidney  Double right kidney with malrotation |  | normal | >25 years,  normal renal function |
| **19 S1** | **M** | - BMF age 8 - Hypothyroidism - mild microcephaly - short stature | Asian | Y | FANCA | 10 years (sibling) | HSCT age 10 | Both kidneys normal |  | normal | 8 years  normal renal function |
| **20 S2** | **M** | - BMF age 4 - Hypospadias - short stature - hypoplastic L thumb | Asian | Y | FANCA | 8 years | HSCT age 10 | Both kidneys normal |  | normal | 8 years  normal renal function |
| 21 | **F** | - BMF - Short stature - Duodenal atresia | White British | N | *FANCA* | 4 years | Small bowel corrective surgery as infant  HSCT age 4 | Bilateral dysplastic kidneys |  | AKI  post HSCT | 8 years  mildly elevated creatinine  eGFR 20-40 mls/min/1.73m^2^ |
| 22 | **F** | - Short stature - Duplex thumb - Bone marrow failure | White British | N | *FANCA* | 8 months | Awaiting HSCT | Atrophic left cystic kidney (nephrectomised)  Normal right kidney | UTI as infant  DMSA: non-functioning left kidney | normal | 3 years  normal renal function |
| 23 | **M** | - Polydactyly right hand - Bone marrow hypoplasia - Hypoplastic ear canal - Short stature | White British | N | *FANCA* | 5 months |  | Left Pelvic kidney  Right kidney hydro-nephrosis | UTI as infant  MCUG  (vesiculo-uretheral reflux grade II right kidney, trabeculated bladder, no PUV)  DMSA (17% function left pelvic kidney) | normal | 2 years  normal renal function |
| 24 | **F** | - Prematurity 32 wks - Hypoplastic right thumb - Abnormal genitalia with absent clitoris and labia minora - Failure to thrive - Short stature - Absent ovaries | White British | N | *FANCI* | 3 years | HSCT awaited | Both kidneys normal |  | Normal | 2 years  normal renal function |
| 25 | **F** | - Short stature - Ovarian failure - Hypothyroidism - BMF | White British | N | *undetermined* | 9 years | BMF age 10  HSCT age 14 | Multi cystic left kidney  Normal right kidney |  | normal | 21 years  normal renal function |
| 26 | **M** | - Short stature - FTT - BMF | White British | N | *FANCA* | 9 years | BMF age 9  HSCT age 11 | Both kidneys normal |  | normal | 23 years  normal renal function |
| 27 | **F** | - Borderline microcephaly - BMF | White British | N | *FANCA* | 7 years | HSCT age 10  SCC epiglottitis age 27 | Both kidneys normal |  | normal | 15 years  normal renal function |
| 28 | **F** | - Borderline microcephaly - Short statue - BMF | White British | N | *FANCA* | 8 years | HSCT age 9 | Right Duplex Kidney  Left kidney normal |  | normal | 8 years  normal renal function |
| 29 | **M** | - Borderline microcephaly - Borderline short stature - BMF | Mixed | N | undetermined | 5 years | Severe BMF  HSCT age 5 | Malrotation left kidney  Normal right kidney |  | Normal | 3 years  normal renal function |
| 30 | **F** | - Microcephaly - FTT - Short stature - BMF | White British | N | *FANCD2* | 9 years | HSCT age 10 | Both kidneys normal |  | normal | 9 years  Normal renal function |

**Supplemental Table 1**: Clinical and genetic details of FA-patient cohort studied. Mild phenotype is not shaded at the bottom, classic in light grey and severe is in dark grey boxes.

C/S: consanguinity. BMF: Bone marrow failure. Sibling pairs with bold patient numbers as indicated.

Abbreviations:

AML Acute myeloid leukaemia

BMF Bone marrow failure

CHD Congenital Heart disease

FTT Failure to thrive

HSCT Hematopoietic Stem Cell Transplant

IUGR Intrauterine growth retardation

HNSCC Head and neck squamous cell carcinoma

USS Ultrasound scan

DMSA Dimercaptosuccinic acid radio-nuclide scan

MCUG Micturating cysto-urethrogram

MRI Magnetic resonance imaging
